# Impaired Angiogenesis and Th1/Th17 Polarization: A Possible Explanation for the Decreased Incidence of Rosacea in the Aged

**DOI:** 10.1002/iid3.70108

**Published:** 2024-12-18

**Authors:** Juan Long, Zhili Deng, Mengting Chen, Tangxiele Liu

**Affiliations:** ^1^ Department of Dermatology The Affiliated Children's Hospital of Xiangya School of Medicine, Central South University (Hunan Children's Hospital) Changsha China; ^2^ Department of Dermatology Xiangya Hospital, Central South University Changsha China

**Keywords:** angiogenesis, incidence, LL37, rosacea, Th1/Th17 polarization

## Abstract

**Background:**

Rosacea is a common inflammatory skin disorder characterized by frequent facial flushing, erythema, telangiectasia, and papules, with a higher incidence observed in individuals aged 30−50 years and a tendency to decrease in the elderly. This age‐related decline in incidence drew our attention to further explore the relationship between rosacea pathogenesis and aging.

**Methods:**

We analyzed the incidence of rosacea across 8340 individuals without systemic diseases. The effects of LL37‐induced rosacea‐like erythema and inflammation were evaluated in both young and aged mice. Immunofluorescence analysis was performed to assess microvessel density, whereas the expression levels of angiogenesis‐related factors, including matrix metalloproteinases (MMPs) and vascular endothelial growth factor α (VEGFα), were quantified. Additionally, immune responses were assessed at both the cellular and systemic levels.

**Results:**

Aged mice displayed milder LL37‐induced rosacea‐like erythema and inflammation compared to their young counterparts. Immunofluorescence analysis revealed a decrease in microvessel density in rosacea models of the aged group. The expression of angiogenesis‐related factors, including MMPs and VEGFα, was decreased in aged mice compared to young mice, indicating a reduced responsiveness to LL37 stimulation. Furthermore, we found that suppressed Th1‐ and Th17‐polarized immune responses, one of the major pathogenic mechanisms of rosacea, were reduced in aged mice in response to LL37 stimulation at both cellular and systemic levels.

**Conclusion:**

The findings suggest that impaired angiogenesis and attenuated Th1/Th17 immune responses underlie the age‐related decline in rosacea incidence.

## Graphical abstract

This study explores the impact of aging on rosacea, an inflammatory skin disorder that commonly affects individuals aged 30−50. Our findings indicate that aged mice exhibit milder rosacea‐like symptoms, a decreased microvessel density, and reduced expression of angiogenesis‐related factors and attenuated Th1/Th17 immune responses. These results suggest that impaired angiogenesis and a diminished immune response contribute to the lower incidence of rosacea in the elderly.

## 1 Introduction

Rosacea is a chronic inflammatory skin condition characterized by recurrent remissions and exacerbations. Its prevalence varies significantly across geographic regions, with rates reported between 1% and 20% [1, 2]. A slightly lower rate of 3.48% has been observed among the Chinese population [3]. Epidemiological studies suggest that the peak incidence occurs between the ages of 30 and 50. Notably, the incidence of rosacea shows a declining trend with increasing age [3, 4, 5]. This age‐related decline in incidence drew our attention to further explore the relationship between rosacea pathogenesis and aging.

Despite significant advances in research, the etiology and underlying mechanisms of rosacea remain insufficiently understood. Recent findings highlight the roles of dysregulated innate and adaptive immunity, complex neuroimmune interactions, impaired neurovascular function, microbial colonization, compromised skin barrier integrity, and genetic susceptibilities to the pathogenesis of rosacea [6, 7]. Intradermal injection of the peptide LL37 in murine models has been shown to trigger inflammatory cascades, resulting in erythema and telangiectasia—hallmarks of rosacea‐like dermatitis [8, 9]. LL37 is cleaved from cathelicidins by the kallikrein‐related peptidase (KLK) family, primarily KLK5, activating pathways that lead to the release of inflammatory mediators, including matrix metalloproteinases (MMPs), specifically MMP2 and MMP9, which play critical roles in extracellular matrix degradation [10, 11, 12, 13]. Moreover, LL37 promotes the release of cytokines and chemokines during immune responses, demonstrating chemotactic effects on various immune cells. Recent research underscores the significance of T‐cell responses in rosacea lesions, predominantly driven by Th1/Th17 cell polarization [14, 15, 16].

Thus, this study aims to elucidate the intrinsic relationship between aging and the development of rosacea, focusing specifically on the potential underlying mechanisms.

## 2 Results

### 2.1 Aging Alleviates the LL37‐Induced Rosacea‐Like Erythema and Inflammation

**Figure 1:**
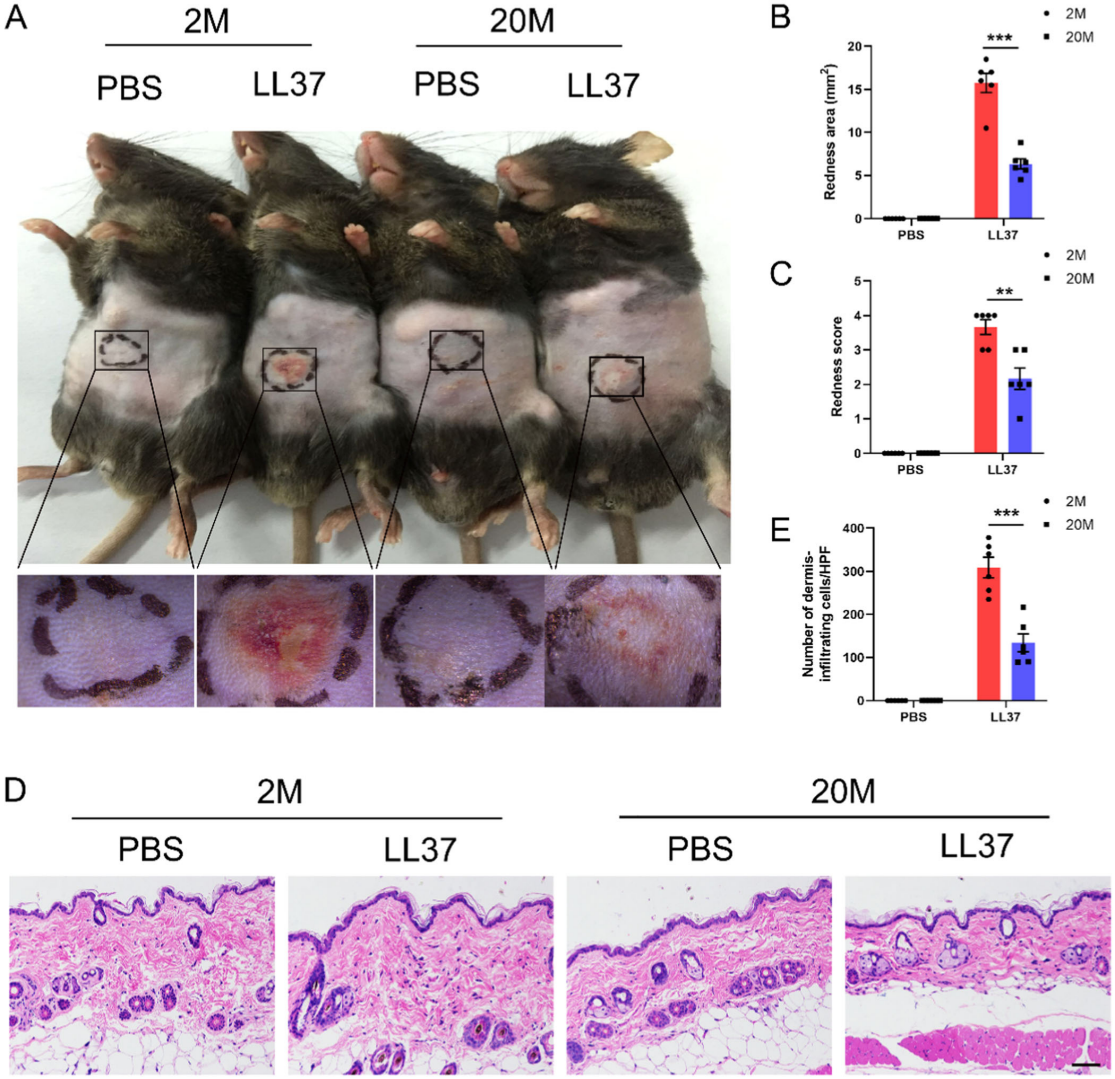
Aging alleviates LL37‐induced rosacea‐like erythema and inflammation. (A) Back skin images from young (2 months, 2M) and aged (20 months, 20M) mice intradermally injected with LL37 or control vehicle (*n* = 6 per group), captured 48 h postinjection. Below are magnified images of the boxed regions. The severity of the rosacea‐like phenotype was assessed based on the (B) area and (C) score. (D) HE staining of skin lesions from young and aged mice treated with LL37 or vehicle; scale bar = 100 μm. (E) Dermal infiltrating cells in (D) were quantified using ImageJ. All results are representative of at least three independent experiments. Data represent the mean ± SEM. **p < 0.01, ***p < 0.001. One‐way ANOVA with Bonferroni's post hoc test was used (B, C, and E).

In a comprehensive study at the Physical Examination Center of Xiangya Hospital, we analyzed a cohort of 8340 individuals free of systemic diseases, stratifying them into four age groups: < 25, 25−44.9, 45−64.9, and ≥ 65 years. Our results indicated that the highest incidence of rosacea was in the 25−44.9 age group (4.5%), with a notable decline to 2.9% in the 45−64.9 age group and 0.9% in individuals aged 65 and older (Table 1). These findings strongly suggest that aging is a significant factor influencing the pathogenesis of rosacea. To further investigate this relationship, we employed a validated animal model in which intradermal LL37 injection induced an inflammatory response characteristic of rosacea [17]. Young mice displayed pronounced rosacea‐like features that escalated with successive doses, whereas aged mice showed milder erythema, lighter coloration, and reduced area involvement (Figure 1A). Quantitative assessments revealed significant reductions in both the average redness area and severity scores in aged mice (Figure 1B,C). Histological analysis revealed that, following multiple injections of LL37, aged mice had significantly less inflammatory cell infiltration within the dermis compared to young mice (Figure 1D,E). These observations highlight the attenuation of LL37‐induced rosacea‐like features with aging, illuminating the complex interplay between age‐related elements and the emergence of rosacea‐like phenotypes.

**Table 1:** The incidence of rosacea in different age groups.

	No of cases (%)		
Characteristics	Overall	Females	Males
Age category			
< 25	4 (2.3)	2 (2.3)	2 (2.3)
25−44.9	113 (4.5)	89 (6.0)	24 (2.4)
45−64.9	136 (2.9)	112 (4.3)	24 (1.2)
≥ 65	8 (0.9)	5 (1.3)	3 (0.5)
Total	261 (3.1)	208 (4.5)	53 (1.4)

### 2.2 Aging Is Associated With a Diminished Expression of Rosacea‐Related Signature Genes

**Figure 2:**
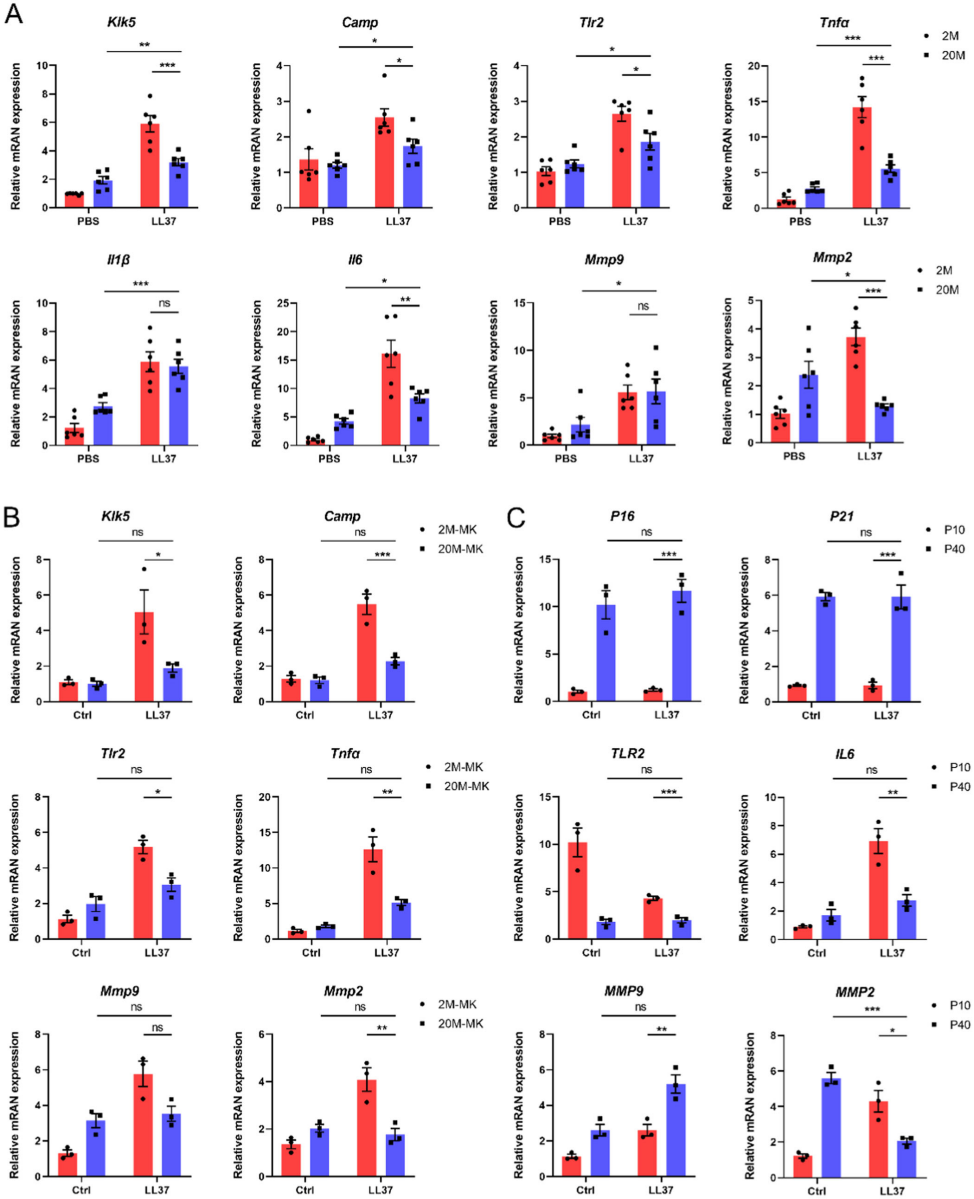
Aging is associated with a diminished expression of rosacea‐related signature genes. (A) Relative mRNA levels of *Klk5*, *Camp*, *Tlr2*, *Tnfα*, *Il1β*, *Il6*, *Mmp9*, and *Mmp2* were measured in the lesional skin of young and aged mice treated with LL37 or control vehicle (*n* = 6 per group). (B) MKs isolated from young and aged mice were treated with LL37 for 24 h. mRNA levels of *Klk5*, *Camp*, *Tlr2*, *Tnfα*, *Mmp9*, and *Mmp2* were quantified by RT‐qPCR. (C) HDFs from P10 and P40 were similarly treated, quantifying *P16*, *P21*, *TLR2*, *IL6*, *MMP9*, and *MMP2* levels. All results are representative of at least three independent experiments. Data represent the mean ± SEM. *p < 0.05, **p < 0.01, ***p < 0.001, ns indicates no significance. One‐way ANOVA with Bonferroni's post hoc test was used.

Elevated levels of disease characteristic factors, such as KLK5, CAMP, TLR2, and proinflammatory cytokines (such as TNF‐α, IL1β, and IL6), and MMPs have been observed in the skin lesions of human rosacea patients as well as in a mouse model of rosacea induced by LL37 [6, 14, 18]. Our data indicate that mRNA levels of these rosacea‐related signature genes were significantly increased in the skin lesions of young mice after multiple LL37 injections. However, this upregulation was not evident in aged mice compared to young mice. Notably, there was even a reversal of the trend of high MMP2 expression in the aged group (Figure 2A). To further elucidate the cellular implications of aging in the pathogenesis of rosacea, we isolated primary mouse keratinocytes (MKs) from both young and aged mice and treated them with LL37 in vitro. We found that LL37 significantly increased the expression of *Klk5*, *Camp*, *Tlr2*, *Tnfα*, *Mmp9*, and *Mmp2* in young MKs, exceeding the levels observed in aged MKs (Figure 2B). Similarly, human dermal fibroblasts (HDFs) with over 40 passages (> P40) exhibited diminished responsiveness to LL37 compared to younger counterparts (Figure 2C). Consistent with skin lesions, the LL37‐induced MMP2 increase in both MKs and HDFs showed a reversal trend in aged cells.

### 2.3 Alleviation of Rosacea in Aged Mice May Be Associated With Impaired Angiogenesis

**Figure 3:**
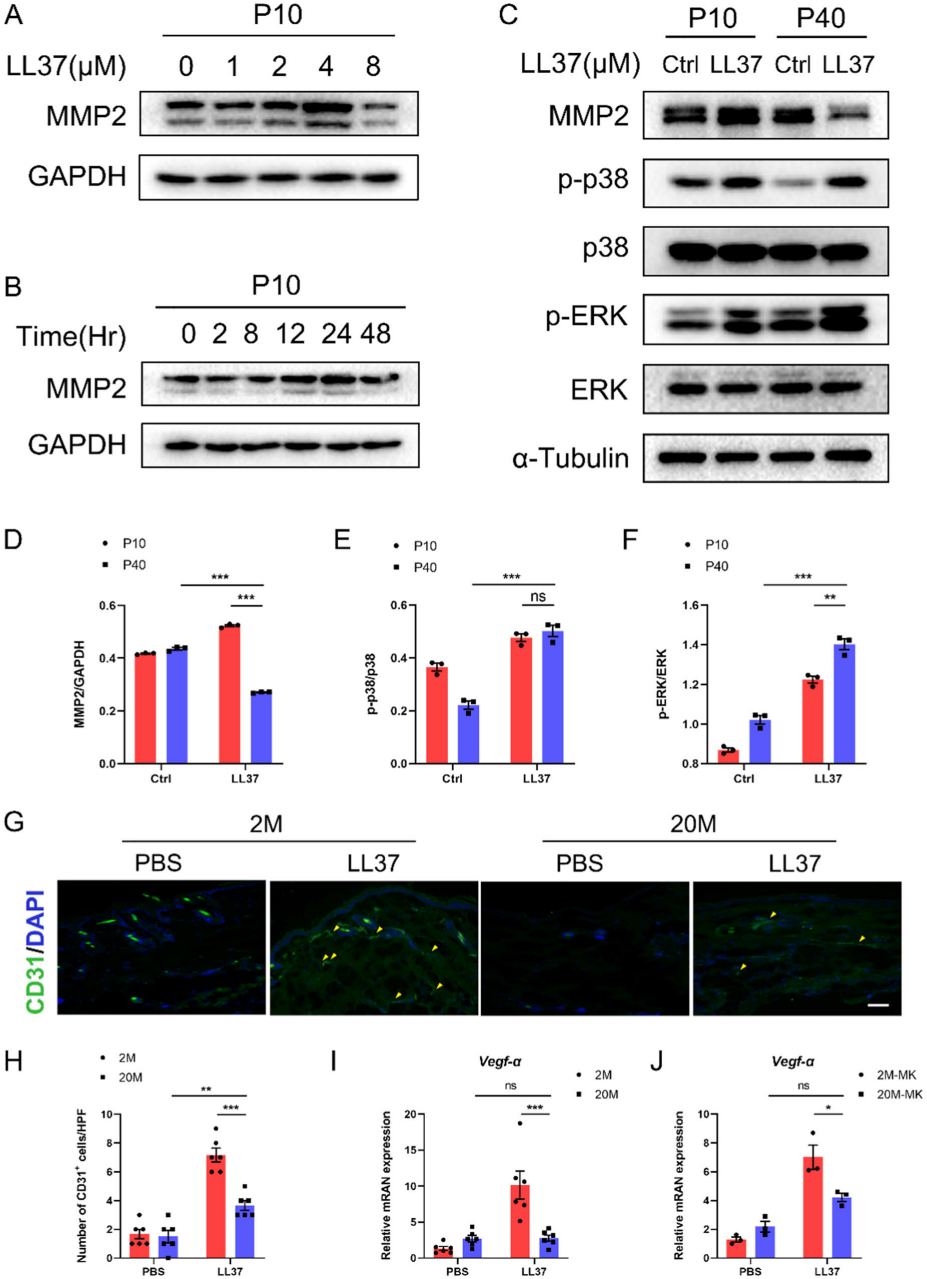
Alleviation of rosacea in aged mice may be associated with impaired angiogenesis. (A) Immunoblot of MMP2 expression levels in young HDFs treated with different doses of LL37 (0–8 μM). (B) Immunoblot of MMP2 expression levels in young HDFs treated with different times of LL37 (0–48 h). (C) Immunoblot of p‐p38, total p38, p‐ERK, and total ERK in young and aged HDFs treated with LL37. Relative quantification of protein levels of (D) MMP2, (E) p‐p38, and (F) p‐ERK. (G) Representative immunofluorescence images showing CD31 (green) expression of lesional skin sections from young and aged mice treated with LL37 or PBS. DAPI staining (blue) indicates nuclear localization. Scale bar, 100 μm. (H) The number of dermal CD31‐positive cells was quantified for lesional skin sections from young and aged mice treated with LL37 or PBS. (I) The relative mRNA levels of *Vegfα* in lesional skins from young and aged mice treated with LL37 or PBS. (J) The relative mRNA levels of *Vegfα* in young and aged MKs treated with LL37 or control vehicle. All results are representative of at least three independent experiments. Data represent the mean ± SEM. *p < 0.05, **p < 0.01, ***p < 0.001. ns indicates no significance. One‐way ANOVA with Bonferroni's post hoc test was used.

Flushing and burning sensations on the central face are hallmarks of rosacea, primarily attributed to abnormal angiogenesis and vascular hyper‐reactivity [19, 20]. MMPs serve as key inflammatory mediators linking the processes of inflammation with angiogenesis and vascular remodeling [21]. Previous studies have shown that the expression of MMP2 and MMP9 is increased in rosacea skin [12]. Our earlier results indicated a reduction in MMP2 mRNA levels in the skin lesions of aged mice. At the cellular level, we observed that LL37 failed to increase MMP2 expression in aged MKs and HDFs. We subsequently assessed MMP2 protein levels. LL37 treatment in young HDFs resulted in a concentration‐ and time‐dependent increase in MMP2 protein levels, whereas aged HDFs displayed a lack of responsiveness (Figure 3A,B). The regulation of MMPs is mediated by a complex network of interconnected signaling pathways. We analyzed phosphorylated forms of MAPK pathway molecules, specifically P‐p38 and P‐ERK. LL37 significantly elevated p38 and ERK phosphorylation in young HDFs, yet in aged cells, despite an increase, this did not correlate with MMP2 expression trends (Figure 3C–F). These results suggest that the alleviation of rosacea symptoms in aged mice may be related to the inability of LL37 to upregulate MMP2 and that this is not directly mediated by the MAPK pathway. Furthermore, we explored the impact of aging on rosacea‐like angiogenesis. Immunostaining for CD31, a blood vessel marker, revealed that LL37 treatment significantly increased CD31^+^ blood vessels in young mice, a response markedly attenuated in aged counterparts (Figure 3G,H). Elevated levels of vascular endothelial growth factor (VEGF) have been reported in the lesional skin of rosacea patients compared to healthy controls [22]. Notably, LL37 failed to induce VEGF elevation in aged mice (Figure 3I). In vitro experiments corroborated that LL37 specifically induces upregulation of *Vegfα* in young MKs (Figure 3J). Collectively, these results suggest that the reduced vascular reactivity to LL37 stimulation in aged mice may contribute to the attenuation of rosacea‐like inflammation and erythema.

### 2.4 Attenuated LL37‐Induced Th1/Th17 Immune Responses Underlie Age‐Related Remission of Rosacea

**Figure 4:**
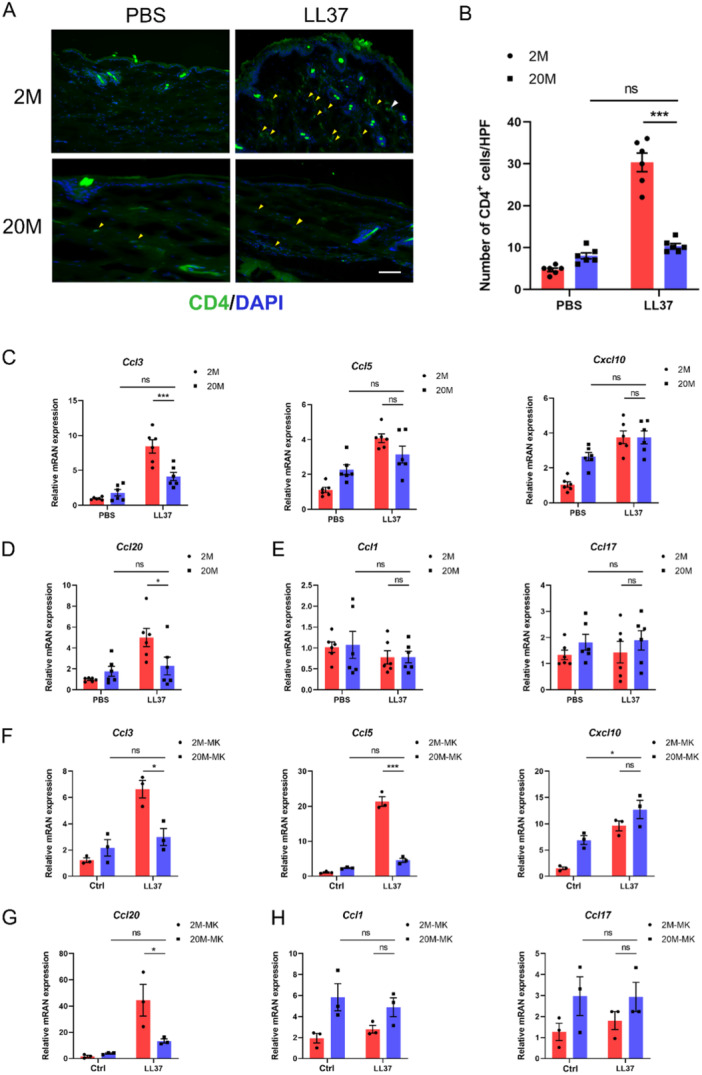
Attenuated LL37‐induced Th1/Th17 immune responses underlie age‐related remission of rosacea. (A) Representative immunofluorescence images showing CD4 (green) expression of skin sections from young and aged mice treated with LL37 or PBS. DAPI staining (blue) indicates nuclear localization. Scale bar, 100 μm. (B) The number of dermal CD4‐positive T cells was quantified for skin sections from young and aged mice treated with LL37 or PBS. (C) Relative mRNA levels of *Ccl3*, *Ccl5*, and *Cxcl10* in skins from young and aged mice treated with LL37 or PBS (*n* = 6 for each group). (D) Relative mRNA levels of *Ccl20* in skins from young and aged mice treated with LL37 or PBS. (E) Relative mRNA levels of *Ccl1* and *Ccl17* in skins from young and aged mice treated with LL37 or PBS. (F) Relative mRNA levels of *Ccl3*, *Ccl5*, and *Cxcl10* in young and aged MKs treated with LL37 or control vehicle. (G) Relative mRNA levels of *Ccl20* in young and aged MKs treated with LL37 or control vehicle. (H) Relative mRNA levels of *Ccl1* and *Ccl17* in young and aged MKs treated with LL37 or control vehicle. All results are representative of at least three independent experiments. Data represent the mean ± SEM. *p < 0.05, **p < 0.01, ***p < 0.001. ns indicates no significance. One‐way ANOVA with Bonferroni's post hoc test was used.

Previous studies have shown that CD4^+^ T cells are integral to the pathophysiology of rosacea, characterized by an increased abundance of CD4^+^ T cells and polarization toward Th1/Th17 cell phenotypes within rosacea skin lesions [14, 23]. To investigate whether the reduced incidence of rosacea in the elderly is associated with a similar imbalance in adaptive immunity, we measured dermal CD4^+^ T cell populations using immunofluorescence. Consistent with observations in rosacea patients, we found a marked increase in infiltrating CD4^+^ T cells in the skin lesions of young mice with LL37‐induced rosacea‐like dermatitis, whereas fewer CD4^+^ T cells were infiltrated in aged mice (Figure 4A,B). Additionally, our results showed that LL37 induced significant upregulation of Th1 polarization‐related genes *Ccl3*, *Ccl5*, and *Cxcl10*, and Th17 polarization‐related gene *Ccl20* in young mice but not in aged mice (Figure 4C,D). The expression levels of Th2 polarization‐related genes *Ccl1* and *Ccl17* were not significantly increased in the skin lesions of either young or aged mice (Figure 4E). Similar patterns were observed in MKs (Figure 4F–H). Taken together, these findings suggest that the alleviation of rosacea symptoms in aged mice may be correlated with a decreased polarization of Th1/Th17 cells, which subsequently leads to a decreased accumulation of pathogenic Th1 and Th17 cells in skin lesions.

## 3 Discussion

Rosacea is a chronic inflammatory skin disease with a complex pathogenesis. The prevalence and peak incidence of rosacea vary widely, taking into account race, skin type, environment, and lifestyle [2, 3, 24, 25]. The disease typically affects individuals between the ages of 30 and 55 years. In our study, we found an overall incidence of rosacea at 3.1%, with the highest rates observed in individuals aged 25−44 years. Notably, the incidence decreased with advancing age, dropping to 0.9% in individuals aged 65 years and older. In our established mouse model of rosacea‐like inflammation, we observed a progressive increase in both the severity and extent of erythema in young mice following repeated intradermal injections of LL37; however, aged mice exhibited no significant erythema. Our experiments at both the system and cellular levels revealed that aging alleviates the Th1/Th17 polarization‐related inflammation and angiogenesis in rosacea.

Aging exerts a suppressive effect on immune cell function, leading to a systemic weakening of innate and adaptive immune defenses [26, 27]. The aberrant function of TLR2 and the antimicrobial peptide LL37 leads to dysregulation of the innate immune system and promotes a downstream inflammatory cascade as an important mechanism in the pathogenesis of rosacea. Elevated levels of antimicrobial peptides, KLK5, TLR2, and MMPs have been observed in skin lesional of rosacea patients. MMPs and TLR2 may amplify KLK5 activity, facilitating the conversion of antimicrobial peptides into their active form, LL37, which in turn exacerbates inflammation and angiogenesis [28, 29]. In the present study, we found that the expression of these factors was not as markedly upregulated in aged mice as in young mice. These findings indicate a compromised ability of the aging immune system to mount effective responses to pathogens and external stimuli. This result is consistent with previous findings that the decline of the TLR2/NF‐κB signaling pathway in aged mice is involved in the insensitive immune response [30].

MMPs play a pivotal role in degrading extracellular matrix components and facilitating leukocyte migration, integral for skin tissue remodeling. The clinically effective treatment of rosacea with low‐dose doxycycline operates by inhibiting MMP activation through indirect suppression of keratinocyte MMP expression [31]. In keratinocytes and fibroblasts, MMPs can be induced by UV light and reactive oxygen species stimulation, which may also be a factor in the triggering or exacerbation of rosacea. Furthermore, increasing evidence supports that MMPs contribute to angiogenesis and remodeling by degrading vascular basement membrane components and modulating angiogenic growth factors and cytokines [32]. Aging and cellular senescence disrupt vascular mechanotransduction, leading to impaired cell responses to hemodynamic changes. The accumulation of senescent endothelial and smooth muscle cells, along with senescence‐associated secretory phenotypes in aging vessels, impairs angiogenesis and contributes to microvascular rarefaction and pathological extracellular matrix remodeling [33]. This may partly explain the diminished angiogenesis and the reduced expression of VEGF and MMPs observed in aged mice following LL37 stimulation. Interestingly, in our current study, compared to the control group injected with PBS, the levels of LL37‐induced inflammatory factors and angiogenic factors were elevated in aged mice, although not as markedly as in young mice. However, the expression of MMP2 was reduced in aged mice. Similarly, at the cellular level, we observed that LL37 failed to induce an increase in MMP2 in aged dermal keratinocytes and fibroblasts. Given the important role of the MAPK pathway in inflammation mediated by the antimicrobial peptide LL37 [34], we explored whether the downregulation of MMP2 in senescent fibroblasts is regulated by this signaling pathway. Subsequently, the results indicate that the observed changes in MMP2 expression may not be directly mediated by the MAPK pathway. However, the underlying mechanisms remain largely unclear, and further evidence is needed to elucidate their causes.

Previous research has underscored the role of adaptive immune responses in rosacea. Our findings highlight that activated CD4^+^ T cells are predominant, with a notable absence of CD8^+^ T cell involvement. Th1/Th17 polarization‐related inflammation is a key driver in rosacea progression [14, 35]. In our studies, LL37 stimulation did not enhance CD4^+^ T cell infiltration in aged mice. Furthermore, significant differences in gene expression related to Th1/Th17 differentiation (including Ccl3, Ccl5, Cxcl10, and Ccl20) were observed between young and aged groups. In contrast, expression levels of Th2‐related molecules (Ccl1 and Ccl17) showed no significant variation. The observed reduction in rosacea‐like erythema and inflammation in aged mice correlates with decreased Th1/Th17 cell polarization.

Immunosenescence, or immune aging, has been extensively characterized since its introduction in 1964, predominantly affecting the adaptive immune system, particularly T cell‐mediated responses [36, 37]. There is a notable decline in naive CD4^+^ T cells with age, resulting in a constricted T cell receptor (TCR) repertoire and potentially compromising the immune response to novel pathogens and vaccine antigens [38, 39]. During senescence, there is a noted decrease in Th1 cells and an increase in Th2 cells, leading to an imbalance in the Th1/Th2 ratio. The mammalian target of rapamycin complex 2 (mTORC2) regulates the differentiation of CD4^+^ T cells toward a Th2 phenotype, with its expression levels elevated in aging CD4^+^ T cells. mTORC2 modulates Th2 differentiation by inhibiting suppressors of cytokine signaling, thereby contributing significantly to the observed Th1/Th2 imbalance [40, 41]. Additionally, the expression levels of aquaporins (AQPs) in the skin decrease with aging, particularly for AQP3, a channel protein that mediates the transport of water and glycerol. Our previous study revealed that AQP3 is highly expressed in the epidermis and CD4^+^ T cells of patients with rosacea and experimental mice. The deletion of AQP3 effectively inhibited the development of rosacea‐like skin inflammation in LL37‐induced murine models, suggesting its pivotal role in disease pathogenesis. Notably, AQP3 expression was upregulated during T‐cell differentiation, potentially facilitating Th17 differentiation through the activation of STAT3 signaling [42, 43]. These findings provide insights into the dampening effects of aging on Th1/Th17 immune responses.

Similar characteristics are observed in other dermatological conditions driven by Th1/Th17 pathways, such as psoriasis. Psoriasis is traditionally classified into early onset (before age 40, accounting for 70% of cases) and late onset (at or beyond age 40). Interestingly, late‐onset psoriasis often exhibits a milder clinical course, characterized by a reduced extent of body surface area involvement and lower incidences of nail and joint complications [44, 45]. This pattern suggests a potential attenuation of psoriasis severity with advancing age, possibly reflecting the intricate interplay of immunological and environmental factors associated with aging. However, studies across diverse populations and geographical regions yield controversial findings, and the role of metabolic disorders and systemic inflammation in the development of late‐onset psoriasis remains inadequately explored.

This study acknowledges several limitations. The LL‐37 injection mouse model, initially established by Yamasaki et al. in 2007, is currently the most widely adopted model for investigating rosacea. However, it primarily represents an acute inflammation model, capturing only a subset of the pathophysiological features associated with rosacea, including erythema, capillary dilation, edema, and inflammatory cell infiltration. Given that rosacea is characterized by recurrent chronic inflammation, recent studies indicate a potential involvement of neurogenic inflammation [46]. The 2‐day LL‐37 injection protocol may not adequately activate neuronal pathways, thus failing to replicate the neurogenic hyperreactivity characteristic of rosacea [47]. Additionally, the lack of clinical severity assessment in this study leaves unresolved the question of whether the severity of rosacea diminishes with age. Future investigations should employ a broader array of models and encompass diverse patient cohorts across various age groups, coupled with comprehensive evaluations of clinical parameters, to facilitate a deeper understanding of the underlying pathophysiological mechanisms.

In summary, through comprehensive analyses of rosacea incidence across different age groups, supplemented by rosacea‐like mouse models and in vitro studies, we have elucidated the intrinsic link between aging and the development of rosacea. Future studies will delve deeper into the specific mechanisms by which impaired angiogenesis and Th1/Th17 polarization contribute to the pathogenesis of this condition. Such insights will be crucial for developing targeted therapeutic strategies for rosacea management, particularly in elderly populations.

## 4 Methods

### 4.1 Subjects

The study received ethical approval from the Ethics Committee of Xiangya Hospital, Central South University. Written informed consent was obtained from all participants. All experimental procedures were conducted in accordance with the principles outlined in the World Medical Association Declaration of Helsinki and the Department of Health and Human Services Belmont Report.

### 4.2 Mouse Model

The LL37 peptide (sequence: LLGDFFRKSKEKIGKEFKRIVQRIKDFLRNLVPRTES) was synthesized and purified by Sangon Biotech (Shanghai, China). Female C57BL/6 wild‐type mice were procured from the Slack Company (Shanghai, China) and allowed to acclimatize for 3 days before the commencement of experiments. To establish the rosacea‐like model, mice received intradermal injections of 40 μL of LL37 (320 μM) twice daily at 12 h intervals for 2 days. Control mice were administered an equivalent volume of PBS. All procedures adhered to the guidelines of the Animal Ethics Committee at Xiangya Hospital, Central South University, and the mice were housed in specific pathogen‐free conditions.

### 4.3 RT‐qPCR

Total RNA was extracted from mouse skin and fibroblasts using TRIzol reagent (Thermo Fisher Scientific). RNA quality was assessed using a NanoDrop spectrophotometer (ND‐2000, Thermo Fisher Scientific). mRNA was then reverse‐transcribed into complementary DNA (cDNA) utilizing the Maxima H Minus First Strand cDNA Synthesis Kit with dsDNase (Thermo Fisher Scientific), following the manufacturer's protocol. Real‐time PCR analysis was conducted using an iTaq Universal SYBR Green Supermix (Bio‐Rad) on a LightCycler 96 (Roche) thermocycler. Relative gene expression was quantified using the Δ*C*_t_ method with normalization to GAPDH as the reference gene. The primer sequences for the target genes utilized in this investigation are provided in Table S1.

### 4.4 Immunofluorescence

Skin sections (8 μm) were fixed in 4% paraformaldehyde for 10 min and then rinsed thrice with PBS. Blocking was performed for 30−60 min using a buffer containing 5% normal donkey serum, 1% BSA, and 0.3% Triton X‐100. Primary antibodies were incubated overnight at 4°C. Following this, Alexa Fluor 488‐conjugated secondary antibodies (Thermo Fisher Scientific, Waltham, MA) were applied for 30−60 min at room temperature. After washing, DAPI was used for counterstaining. The primary antibodies used were rat anti‐CD31 (1:100; BD Biosciences) and rat anti‐CD4 (1:100; eBioscience).

### 4.5 Cell Culture

HDFs were isolated from foreskin tissue and cultured in DMEM.

### 4.6 Isolation and Culture of MKs

Skin samples were dissected to remove underlying blood vessels, fat, and other tissues, followed by washing with PBS. The samples were then incubated in Dispase I enzyme in a centrifuge tube overnight at 4°C. The epidermal layer was minced into small fragments, digested in 5 mL of 0.25% trypsin at 37°C for 5−7 min, and then the digestion was halted using DMEM with 10% FBS. The resulting cell suspension was filtered to isolate cells, centrifuged, and the supernatant was discarded. The pelleted cells were resuspended in keratinocyte growth medium and seeded into culture dishes.

### 4.7 Protein Extraction and Immunoblotting

Protein extracts were prepared in a loading buffer. Equal amounts of protein were subjected to SDS‐PAGE and subsequently transferred onto polyvinylidene difluoride membranes. The membranes were blocked for 1 h and incubated with primary antibodies diluted in 3% BSA at 4°C overnight. Following incubation with horseradish peroxidase‐conjugated secondary antibodies for 1 h, immunoblots were visualized using a Bio‐Rad imaging system. The primary antibodies employed included the following: rabbit anti‐MMP2 (1:1000; abcam), mouse anti‐GAPDH (1:10,000; Proteintech), rabbit anti‐p38 (1:1000; Cell Signaling), rabbit anti‐phospho‐p38 (1:1000; Cell Signaling), rabbit anti‐ERK1 (1:1000; abcam), rabbit anti‐phospho‐ERK1 (T202) (1:1000; abcam), and mouse anti‐α‐tubulin (1:5000; abcam). Images have been cropped for presentation.

### 4.8 Statistical Analysis

All statistical analyses were carried out using GraphPad Prism 8. Statistical differences were determined by two‐tailed unpaired Student's *t*‐test and one‐way ANOVA with Bonferroni's post hoc test. *p* < 0.05 was considered statistically significant (*p < 0.05, **p < 0.01, ***p < 0.001).

## Author Contributions

J.L. and T.L. performed most of the experiments, analyzed the data, and wrote the manuscript. Z.D. provided technical support and suggestions for the project. T.L. and M.C. conceived the project and supervised the study.

## Ethics Statement

This study received ethical approval from the Ethics Committee of Xiangya Hospital, Central South University. Written informed consent was obtained from all participants. All procedures involving animals were conducted in accordance with the guidelines established by the Animal Ethics Committee at Xiangya Hospital.

## Conflicts of Interest

The authors declare no conflicts of interest.

## Supporting information

Supporting information.

## Data Availability

The authors have nothing to report.
