# Supplementary material for: Impaired Angiogenesis and Th1/Th17 Polarization: A Possible Explanation for the Decreased Incidence of Rosacea in the Aged
Source: Immun Inflamm Dis. 2024 Dec 18;12(12):e70108. doi: 10.1002/iid3.70108 (PMC11653600; doi:10.1002/iid3.70108)
Supplement: Supplementary file 1 — Supporting information. [file IID3-12-e70108-s001.docx]

**Supplementary Table 1**.

| qPCR primers | 5’-3’ | |
| --- | --- | --- |
| Human-*GAPDH*-F | | TGTTGCCATCAATGACCCCTT |
| Human-*GAPDH*-R | | CTCCACGACGTACTCAGCG |
| Human-*P16*-F | | ATGGAGCCTTCGGCTGACT |
| Human-*P16*-R | | GTAACTATTCGGTGCGTTGGG |
| Human-*P21*-F | | CGATGGAACTTCGACTTTGTCA |
| Human-*P21*-R | | GCACAAGGGTACAAGACAGTG |
| Human-*TLR2*-F | | TTATCCAGCACACGAATACACAG |
| Human-*TLR2*-R | | AGGCATCTGGTAGAGTCATCAA |
| Human-*IL6*-F | | CCTGAACCTTCCAAAGATGGC |
| Human-*IL6*-R | | TTCACCAGGCAAGTCTCCTCA |
| Human-*MMP9*-F | | TGTACCGCTATGGTTACACTCG |
| Human-*MMP9*-R | | GGCAGGGACAGTTGCTTCT |
| Human-*MMP2*-F | | TACAGGATCATTGGCTACACACC |
| Human-*MMP2*-R | | GGTCACATCGCTCCAGACT |
| Mouse-*Gapdh*-F | | AGGTCGGTGTGAACGGATTTG |
| Mouse-*Gapdh*-R | | TGTAGACCATGTAGTTGAGGTCA |
| Mouse-*Klk5*-F | | ATGGGCAATGGCTACCCTG |
| Mouse-*Klk5*-R | | GTTCGGTTCCAGAGGGGTT |
| Mouse-*Camp*-F | | GCTGTGGCGGTCACTATCAC |
| Mouse-*Camp*-R | | TGTCTAGGGACTGCTGGTTGA |
| Mouse-*Tlr2*-F | | GCAAACGCTGTTCTGCTCAG |
| Mouse-*Tlr2*-R | | AGGCGTCTCCCTCTATTGTATT |
| Mouse-*Tnfα*-F | | CTGAACTTCGGGGTGATCGG |
| Mouse-*Tnfα*-R | | GGCTTGTCACTCGAATTTTGAGA |
| Mouse-*Il1β*-F | | GCAACTGTTCCTGAACTCAACT |
| Mouse-*Il1β*-R | | ATCTTTTGGGGTCCGTCAACT |
| Mouse-*Il6*-F | | TAGTCCTTCCTACCCCAATTTCC |
| Mouse-*Il6*-R | | TTGGTCCTTAGCCACTCCTTC |
| Mouse-*Mmp9*-F | | CTGGACAGCCAGACACTAAAG |
| Mouse-*Mmp9*-R | | CTCGCGGCAAGTCTTCAGAG |
| Mouse-*Mmp2*-F | | CAAGTTCCCCGGCGATGTC |
| Mouse-*Mmp2*-R | | TTCTGGTCAAGGTCACCTGTC |
| Mouse-*Vegfα*-F | | CTGCCGTCCGATTGAGACC |
| Mouse-*Vegfα*-R | | CCCCTCCTTGTACCACTGTC |
| Mouse-*Ccl1*-F | | GGCTGCCGTGTGGATACAG |
| Mouse-*Ccl1*-R | | AGGTGATTTTGAACCCACGTTT |
| Mouse-*Ccl3*-F | | TTCTCTGTACCATGACACTCTGC |
| Mouse-*Ccl3*-R | | CGTGGAATCTTCCGGCTGTAG |
| Mouse-*Ccl5*-F | | GCTGCTTTGCCTACCTCTCC |
| Mouse-*Ccl5*-R | | TCGAGTGACAAACACGACTGC |
| Mouse-*Ccl17*-F | | GACGACAGAAGGGTACGGC |
| Mouse-*Ccl17*-R | | GCATCTGAAGTGACCTCATGGTA |
| Mouse-*Ccl20*-F | | GCCTCTCGTACATACAGACGC |
| Mouse-*Ccl20*-R | | CCAGTTCTGCTTTGGATCAGC |
| Mouse-*Cxcl10*-F | | CCAAGTGCTGCCGTCATTTTC |
| Mouse-*Cxcl10*-R | | GGCTCGCAGGGATGATTTCAA |
